# Polyarthritis following a streptococcal infection, a doctor's dilemma in treatment: a case report

**DOI:** 10.1186/1757-1626-2-9140

**Published:** 2009-12-03

**Authors:** Vanya Grover, Robin Dibner

**Affiliations:** 1 Department of Medicine, Lenox Hill Hospital, 100 E 77th Street, New York, NY, 10075, USA

## Abstract

**Introduction:**

Acute Rheumatic Fever and Post Streptococcal Reactive Arthritis in adults can present in a similar manner.

**Case Presentation:**

A 25 year old Caucasian male developed a transient crippling polyarthritis one month following treatment of a sore throat. The patient was found to meet criteria for both Acute Rheumatic Fever and Post Streptococcal Reactive Arthritis, leading to the question of the role of antibiotic prophylaxis long term.

**Conclusion:**

Due to overlap in the diagnostic criteria for Acute Rheumatic Fever and Post Streptococcal Reactive Arthritis in adults, the need for penicillin prophylaxis should be individualized based on exposure risk.

## Background

Acute Rheumatic Fever is a disease state described since the 1800s. It is found most commonly in children, and the disease can manifest in a variety of ways. ARF can present with migratory arthritis involving primarily the large joints, carditis and valvulitis, central nervous system involvement, rash, or any combination of these. These reactions are the sequelae of a pharyngeal infection with group A streptococcus [1].

The Jones criteria were established in 1944 and revised as recently as in 1992 to help make the diagnosis of ARF. The major criteria include carditis, polyarthritis, chorea, erythema marginatum, and subcutaneous nodules. The minor criteria include arthralgia, fever, elevated erythrocyte sedimentation rate, elevated C-reactive protein, or a prolonged PR interval [1]. For diagnosis of ARF, the presence of two major or one major and two minor manifestations and evidence of a preceding group A streptococcal infection is required. A positive throat culture for group A beta-hemolytic streptococci, positive rapid streptococcal antigen test, or elevated or rising streptococcal antibody titer are all acceptable [1].

The treatment for Acute Rheumatic Fever involves antibiotic therapy and anti-inflammatory agents. The use of antibiotic therapy with penicillin is recommended for the treatment of the streptococcal infection, while antibiotic prophylaxis with penicillin, sulfadiazine, or erythromycin is recommended in order to prevent the recurrence of ARF. Anti-inflammatory agents, are used to treat the inflammatory manifestations, and generally produce a dramatic improvement in symptoms at the onset of treatment [1].

The literature cites cases where arthritis following a streptococcal infection was not due to Acute Rheumatic Fever. These cases have been given the diagnosis of Post-Streptococcal Reactive Arthritis (PSReA), first described in 1982 and seen mainly in adults. These patients generally presented with only arthritis, occurring up to ten days after an episode of a streptococcal infection, compared with the average of twenty-one days seen in ARF [2]. They experienced prolonged or recurrent arthritis, lasting an average of two months. The arthritis seen in ARF, in contrast, is found to last one to five days with complete resolution in three weeks [2,3]. PSReA is unresponsive to aspirin or other nonsterioidal medications. Furthermore, extra-articular manifestations, such as tenosynovitis, are often seen in these patients, whereas cardiac involvement is not [2-4].

In children PSReA is readily distinguished from ARF due to the presence of other major Jones Criteria. In adults, differentiating between the two diseases is not as clear. Some patients diagnosed with PSReA also fulfill the one major and two minor criteria for ARF [5]. In regard to treatment the major difference between the two disease states is that antibiotic prophylaxis is not recommended in adults diagnosed with PSReA due to lack of evidence of progression to ARF or development of cardiac sequelae, where it is required in the management of ARF [2].

## Case Presentation

A 25 year old male presented with two weeks of pain and swelling in his knees and ankles. The symptoms began in his right knee, after he experienced minor trauma. He sought emergency room care and was discharged home on ibuprofen after x-rays were negative. The pain and swelling then gradually spread to his left knee and right ankle. At a follow up orthopedic visit medication was continued. His symptoms worsened and impeded his ability to walk.

On day of admission, the patient also complained of new onset of some left MCP and DIP joint tenderness. He denied fevers, chills, shortness of breath, or headaches, but described progressive fatigue over a few weeks. He reported a sore throat six weeks prior, which was treated with amoxicillin for seven days. This was followed by diarrhea which resolved in one week. He was born in the United States, had no medical or surgical history, and no significant family history. He denied the use of cigarettes, alcohol, or illicit drugs. He stated he was currently sexually active with other men. There was no recent travel history, or sick contacts. He worked as a first grade school teacher.

**Figure 1:**
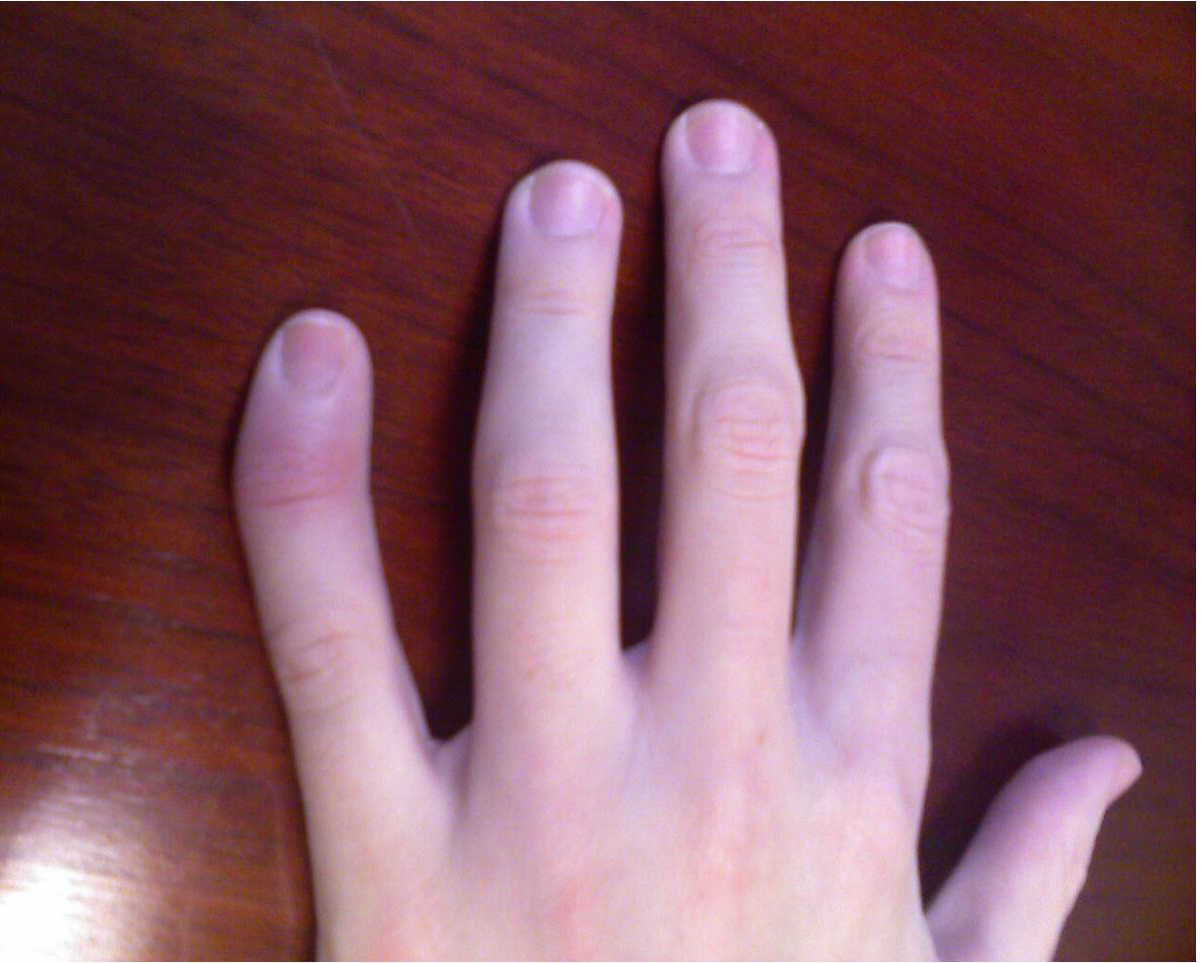
**Periarticular involvement**. Photograph demonstrating periarticular involvement of patient's fifth DIP on the left hand.

On physical exam the patient had a temperature of 100.6F, synovitis and effusions in both knees L>R; right ankle and fifth DIP showed periarticular involvement (Figure 1), tenosynovitis and there was swelling over the dorsum of the left hand. All involved joints were erythematous and exquisitely tender with a decreased range of motion. There was no evidence of a rash, spine tenderness or cardiac murmur.

CBC demonstrated a WBC count of 16.4 (84% polys), platelet count of 1084K, and the ESR was 115. Knee arthrocentesis yielded cloudy yellow fluid (WBC 7,350 with 56% polys), with negative cultures. HIV, urethreal Gonococcal and Chlamydia testing, Rheumatoid Factor, ANA, C. difficile toxins, blood cultures, stool cultures, and throat cultures were negative. The ASLO titer was elevated at 518 (normal <200). EKG demonstrated sinus tachycardia, with a normal PR interval. A transthoracic echocardiogram was unremarkable.

Despite Sulindac 200 mg orally twice daily, the patient's symptoms persisted and a taper of prednisone starting at 40 mg/d orally was required for relief. After discharge he continued to respond well to corticosteroid therapy, and regained strength.

## Discussion

The patient fulfilled the modified Jones criteria for ARF (major: arthritis; minor: fever and elevated ESR), without fitting the typical disease presentation. He also fits many of the criteria for PSReA: adult, arthritis > 3 weeks, no carditis, poor response to NSAID therapy, and absence of other major Jones criteria [5]. As reviewed above, it is difficult to distinguish ARF from PSReA, but necessary in determining the need for antibiotic prophylaxis. This patient would likely be exposed to streptococcus again due to his occupation as a teacher of young children, so our clinical judgment led us to place him on penicillin prophylaxis for at least five years.

## Conclusion

This case exemplifies the overlap between the diagnostic criteria of Acute Rheumatic Fever, and Post Streptococcal Reactive Arthritis. Although these cases are far less common today, they should still be on the differential diagnosis in a patient with arthritis and fever. In an ambiguous case such as this, it is prudent to individualize the antibiotic prophylaxis based on occupational risk or lifestyle.

## Abbreviations

ARF: Acute Rheumatic Fever; PSReA: Post Streptococcal Reactive Arthritis; MCP: Metacarpophalangeal joint; DIP: Distal interphalangeal joint; ANA: Antinuclear Antibody; ASLO: Anti Streptolysin O; NSAID: Non-steroidal anti-inflammatory drug.

## Consent

Written informed consent was obtained from the patient for publication of this case report and accompanying images. A copy of the written consent is available for review by the journal's Editor-in-Chief.

## Competing interests

The authors declare that they have no competing interests.

## Authors' contributions

VG wrote the first draft of the manuscript and RD revised the manuscript. All authors approved the publication of the manuscript.
